# Walking in a dental environment with the “UniCDent” tool kit—part 1: a participatory tool with legs!

**DOI:** 10.3389/froh.2025.1545170

**Published:** 2025-05-23

**Authors:** Prashanti Eachempati, John Martin, Sally Hanks, Mona Nasser

**Affiliations:** ^1^ Peninsula Dental School, University of Plymouth, Plymouth, United Kingdom; ^2^ Faculty of Dentistry, Manipal University College Malaysia, Melaka, Malaysia; ^3^ School of Geography, Earth and Environmental Sciences, University of Plymouth, Plymouth, United Kingdom; ^4^ Faculty of Health, University of Plymouth, Plymouth, United Kingdom

**Keywords:** walking method, participatory research, dental environment, patient perceptions, community or stakeholder engagement

## Abstract

This case study explores the adaptation of the walking method coupled with auto-photography and photo elicitation, a participatory research tool traditionally used in landscape studies, for application in dental research settings. By presenting the UniCDent toolkit, we demonstrate how this immersive method can enhance understanding of patient experiences in dentistry through sensory engagement and spatial exploration. The toolkit comprises four components: Imagery, Gallery Walk, Quadrant Mapping, and Trade-offs, each designed to facilitate active participation and foster in-depth reflections on the dental environment. Participants navigate a simulated dental clinic, documenting their sensory experiences and social interactions, thereby enriching the data collected. This multi-layered approach allows researchers to capture the complexities of patient experiences, providing insights that are applicable across various research projects within dentistry. The commentary highlights the versatility of the walking method, emphasising its role in improving patient-centred care by capturing patient experiences and perspectives. It also demonstrates its value in enhancing the understanding of diverse research enquiries in dental research.

## Introduction

Walking methods are well-established in participatory research. They are commonly utilised in landscape place-based research, allowing participants to engage with their environments through sensory exploration. Walking method encompasses different types, such as exploratory walks (1) walking interviews (2), and transect walks (3) each designed to deepen understanding of the space and its inhabitants (4–7). While predominantly used in outdoor settings—like neighbourhoods and public spaces—these methods have also been applied in indoor environments, including grocery stores and schools (8, 9). Notably, this method has recently found its way into health research, offering valuable insights into the relationship between individuals and their healthcare environments (10). The walking method can be used alongside other participatory approaches, such as auto-photography and photo elicitation, where individuals capture images to reflect their experiences.

However, its application in dentistry remains unexplored, presenting an opportunity for innovation in understanding patient experiences. This article seeks to present a case study on adapting this method to provide an innovative approach to participatory research in dentistry, recognising the significance of spatial factors in shaping patient perceptions within the dental environment. In this case study, we explored how participants experienced and navigated the dental environment using the walking method coupled with auto-photography and photo elicitation. Within the different participatory approaches, the walking method aligns with the Exploration and Visioning domain while also intersecting with Engagement and Capacity Building (11). Auto-photography and photo elicitation fit into the Visual and Narrative domains (12).

In participatory research, the “walking method” immerses participants in a setting to engage their senses and explore firsthand experiences (13, 14). We tailored this method specifically for a dental clinic to address a range of research questions. We demonstrate this method in the context of a specific case study which aims to explore what patients perceive as uncertainty in the dental environment and how they respond to the uncertainty. Instead of relying on traditional interview techniques detached from the environment, the participants journeyed in a simulated dental clinic, allowing them to engage with the setting through their senses—sight, sound, smell, and touch—while also observing the social dynamics between patients and the dental team. This multi-layered approach enhanced participant engagement by anchoring their experiences in the real-world environment, offering richer, more nuanced insights into their interactions and perceptions.

While we used this method to explore uncertainty in the dental clinic, its value lies in its versatility. It can be adapted to a broad range of participatory research initiatives within clinical and other applied settings. By enabling participants to interact directly with their environment, the walking method facilitates a deeper understanding of context-specific phenomena, making it a powerful tool for exploring diverse research questions.

### The toolkit

The UniCDent toolkit, which stands for “Uncertainty in Clinical Dentistry” toolkit, was developed taking inspiration from cult rural tool kit (15). It comprises of four tools that can be used independently or in conjunction with one another, as demonstrated in our participatory research approach. For ease of explaining how the toolkit works or can be applied in practice, we take the specific example of a case study exploring uncertainty in clinical dentistry. This case study was conducted in a multiethno-linguistic population of Malaysia. The study included 12 participants from each ethnic group: Malay, Indian, and Chinese. Maximum variation sampling was used to ensure that each group included individuals with differing levels of uncertainty tolerance.

### Tool one. Imagery: the walking method and auto-photography

**Figure 1:**
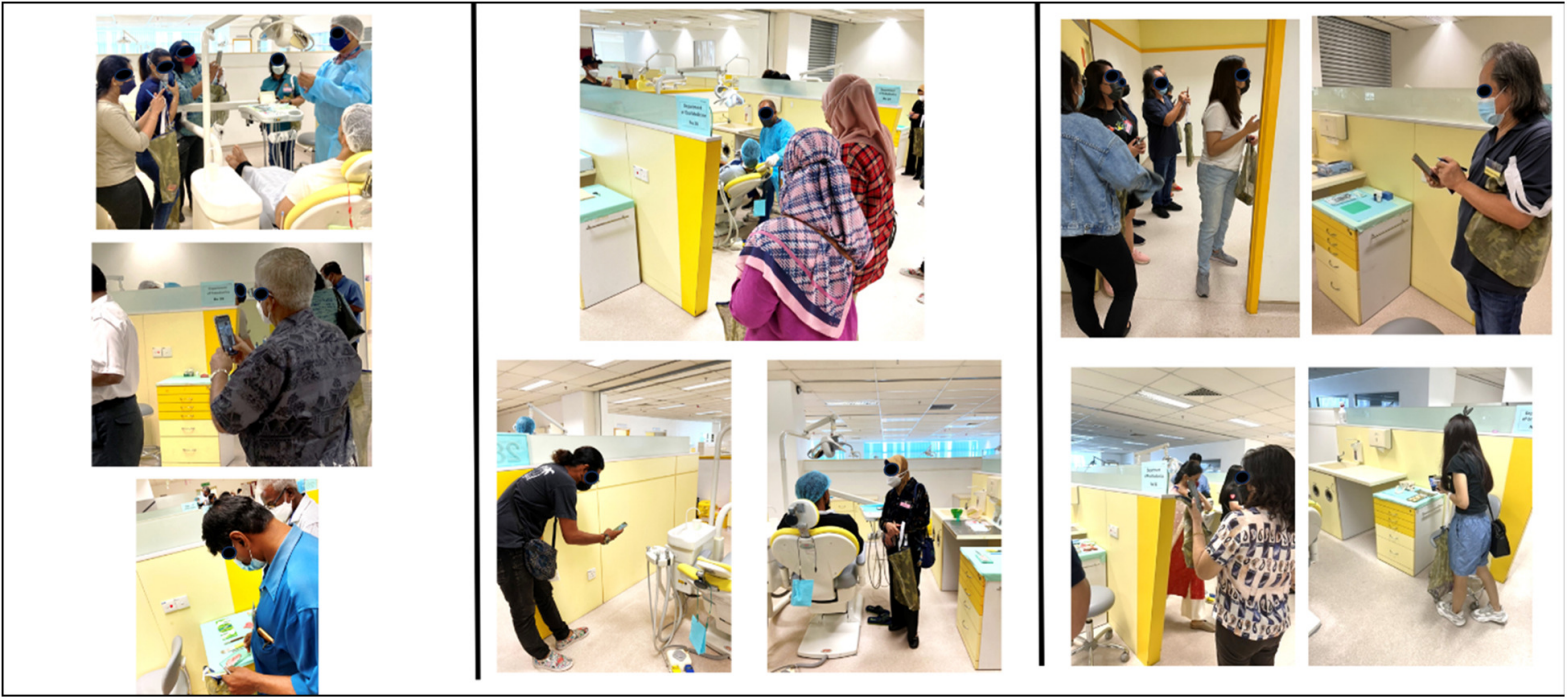
Imagery: participants navigating the dental clinic using auto-photography.

The “*Imagery*” step was central to immersing participants in the dental environment. In this phase, participants journeyed through a simulated dental setup, engaging their senses of sight, smell, touch, and hearing. As they navigated different areas within the clinic, they were encouraged to observe and photograph elements that triggered feelings of uncertainty (Figure 1). They documented not just the visual aspects but also other characteristics of the environment through writing, enhancing their sensory engagement. They were encouraged to select objects and place them in their tote bags as needed to illustrate what triggers uncertainty for them.

This method offered a more immersive and dynamic way of exploring uncertainty compared to traditional interviews that isolate participants from the environment in which their uncertainty arises. By being part of the dental environment, participants could better articulate the specific triggers that evoke uncertainty, giving us a deeper understanding of how these factors interact in a real-world setting.

### Tool two. Gallery walk: exploring photographic interpretations using photo elicitation

**Figure 2:**
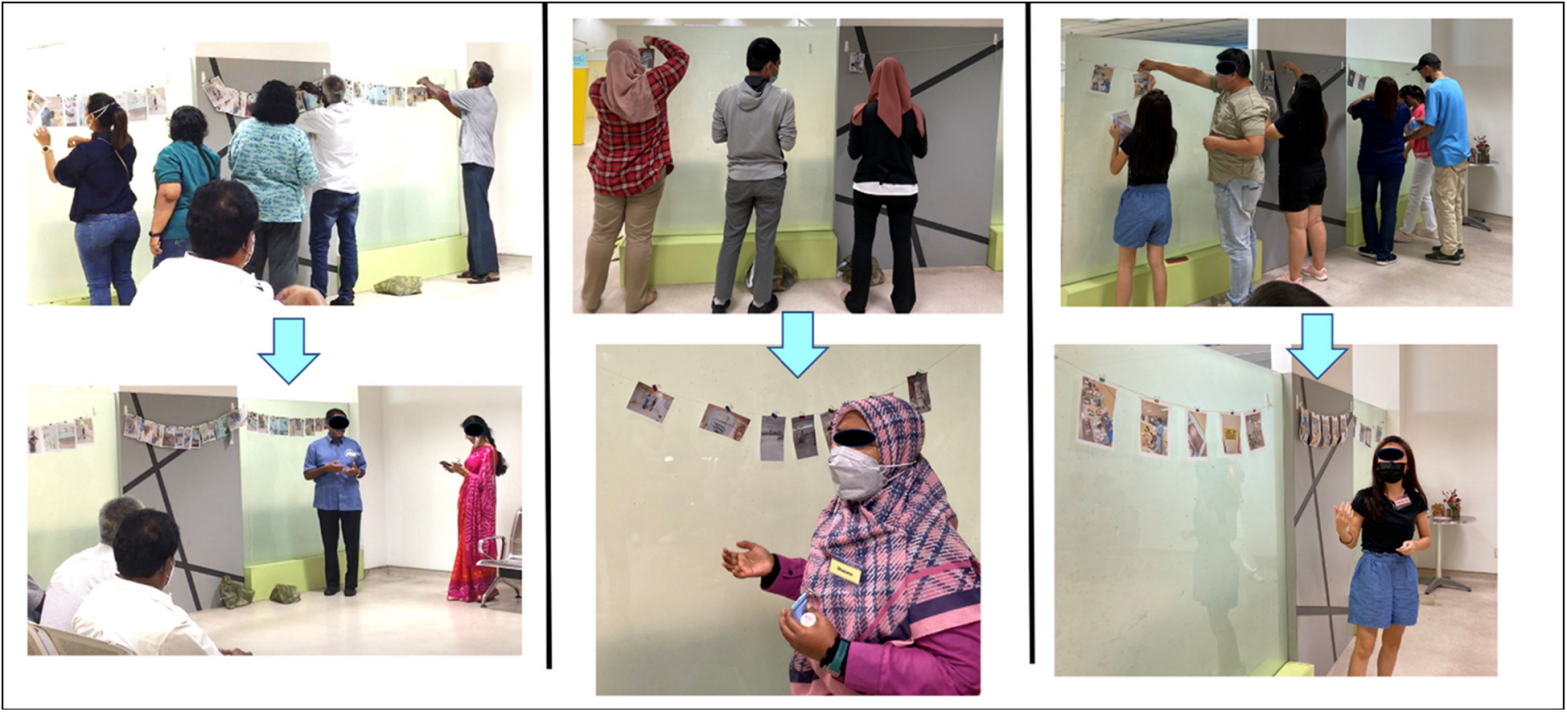
Gallery walk: participants explaining the uncertainty perceived using photo elicitation method.

Following the sensory journey, participants engaged in a “*Gallery Walk”*, where they printed and shared the photographs taken during the tour (Figure 2). This phase explored the subjective meanings participants attributed to their images, offering insight into how specific sights, sounds, or objects contributed to their feelings of uncertainty. The open discussion that followed fostered a collective exploration of these interpretations, as each participant guided the group through their thought process.

Through this activity, we grasped the dynamic and multifaceted nature of uncertainty (16). It became clear that uncertainty is not triggered by isolated factors but by multiple, interacting layers of sensory stimuli and social dynamics. For example, participants noted how the sound of dental burs being used in the handpiece, the smell of dental materials, and the sight of being in a vulnerable position in the dental chair could intensify feelings of anxiety. These sensory elements, coupled with a non-empathetic dentist or an unwelcoming receptionist, further compounded uncertainty, leading participants to question whether they wanted to proceed with treatment, or leave.

This element of the exercise illuminated the significant opportunity to uncover the emotional and psychological complexities that arise in a dental environment. It could be applied to a wide range of research questions, revealing how various elements within a clinical setting influence patient comfort and decision-making. This method enabled us to capture this complexity and provided a platform for patients to articulate how these sensory and social experiences affect their willingness to continue treatment, thus offering deeper insights into the factors that contribute to uncertainty within a dental context.

### Tool three. Quadrant mapping: categorising uncertainty

**Figure 3:**
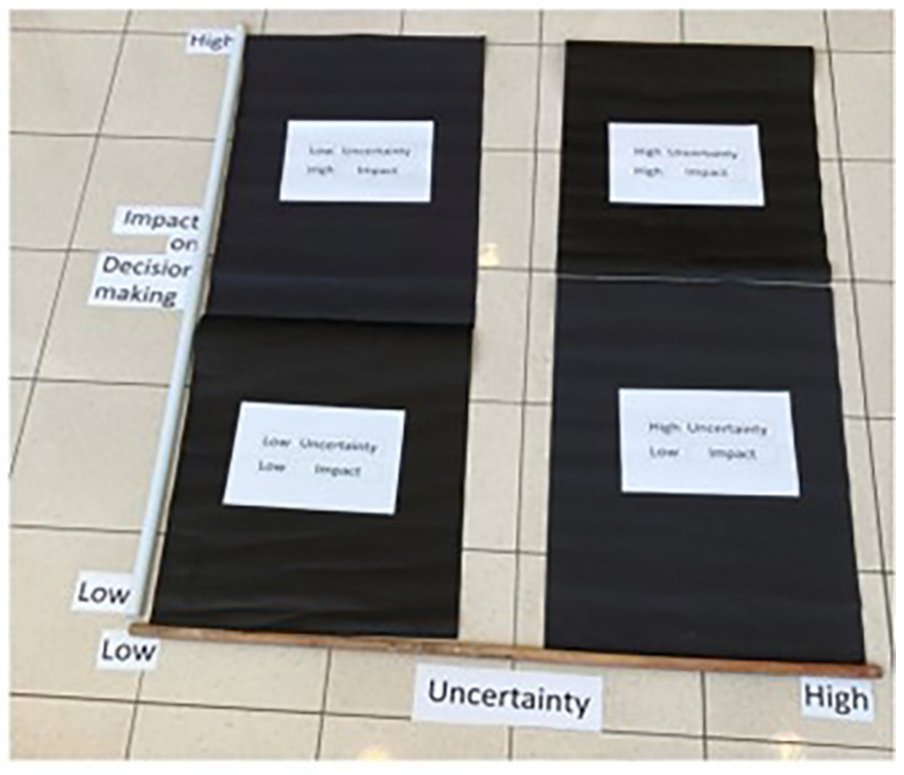
Grid for quadrant mapping.

**Figure 4:**
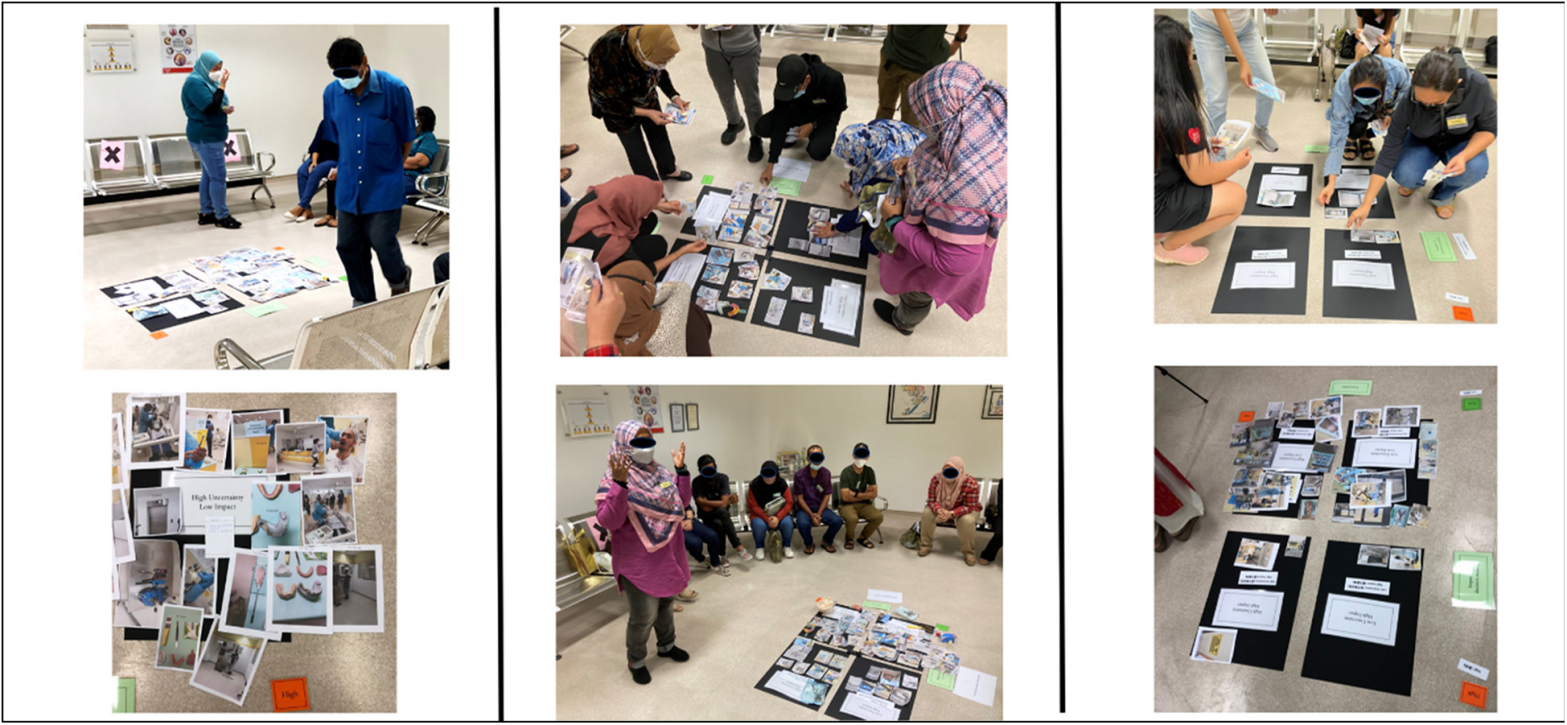
Quadrant mapping: uncertainty classified based on the impact on decision making.

In the “*Quadrant Mapping*” step, a grid with four quadrants was created (Figure 3), where one axis represented the level of uncertainty (High/Low) and the other indicated the impact on decision-making (High Impact/Low Impact). This framework resulted in four quadrants, each representing different combinations: High Uncertainty/Low Impact, High Uncertainty/High Impact, Low Uncertainty/Low Impact, and Low Uncertainty/High Impact. Participants categorised the images and objects they had captured using this grid, based on the level of uncertainty and its perceived impact on their decision-making (Figure 4).

While this structured approach provided clarity on how patients prioritise and weigh their uncertainty in the context of clinical dentistry, it is by no means limited to this application. The framework is flexible and can be customised to suit different research questions and settings, with grid labels adjusted according to the specific focus of interest. What we present here is a case study example to demonstrate its potential, but the quadrant mapping technique can be tailored to suit various participatory research initiatives, allowing for insights specific to the context being studied.

### Tool four. Trade-offs: decision-making when confronted with uncertainty

**Figure 5:**
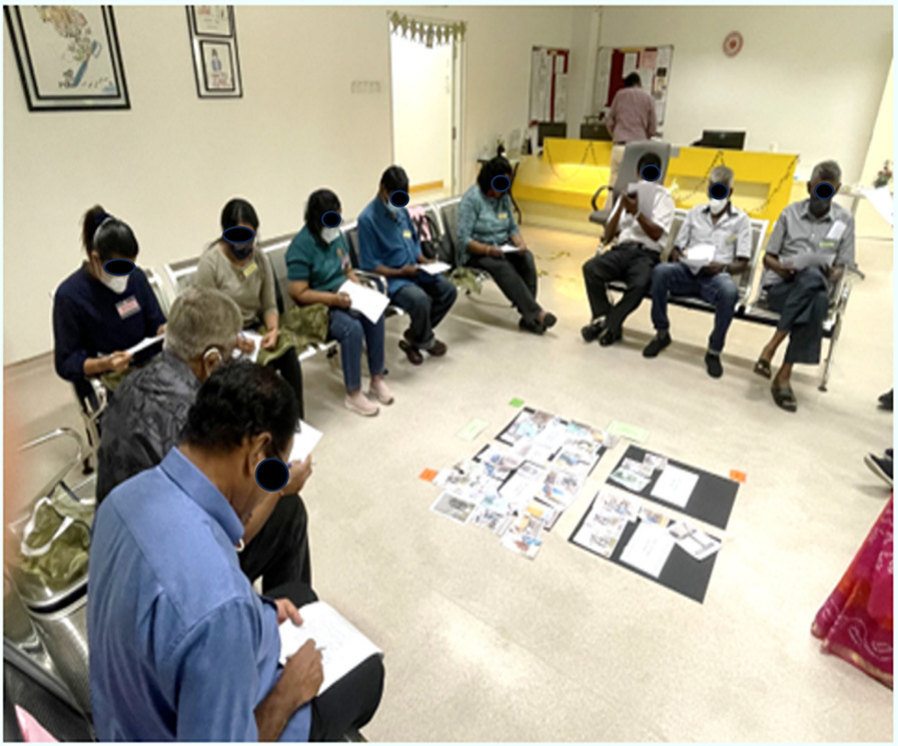
Trade-offs: patients identifying trade-offs when confronted with uncertainty.

The final step, “*Trade-offs*”, explored the factors that influence changes in decision-making when patients experience uncertainty. Participants identified the top three reasons that might prompt them to alter their decisions in a dental setting, particularly in the face of uncertainty. To facilitate this process, participants were given flashcards that listed multiple factors that could influence their decision-making. Empty cards were also provided for them to write in any additional factors they thought of that were not already included in the list (Figure 5). Each participant was instructed to select and rank three flashcards as 1, 2, and 3. Once this ranking was completed, participants were asked to explain their choices.

This exercise revealed the trade-offs patients are willing to make when confronted with uncertainty in clinical decision making. It provided valuable insights into how patients rationalise and navigate their choices when confronted with uncertainty. It highlighted the importance of understanding patients' willingness to compromise and the factors influencing their decisions, as well as how uncertainty shapes their overall dental experience.

Once again, this step can be customised based on the specific research question, as every choice or decision that a patient makes is inherently surrounded by trade-offs. Identifying these trade-offs would be relevant to a variety of research inquiries.

### Insights from this immersive experiment using UniCDent

By adapting the walking method from landscape research (17) to the dental clinic setting, we have developed an immersive participatory tool that extends beyond the study of uncertainty and can be applied to a wide range of research questions.

In our research, the principles of the walking method were applied to explore participants' interactions with the clinical environment (18).

Place referred to how walking through specific locations, such as a dental clinic, activated relationships between participants, the environment, and the sensory elements of that space. As participants moved through the space, they were able to observe the roles of patients, clinicians, and staff, gaining valuable insights into the clinical environment.

Sensory Inquiry emphasised the role of the senses in engaging with the environment. In the context of the dental clinic, participants were absorbed in the setting through the sounds of drills, the smells of materials, and the tactile sensations of instruments. This sensory engagement offered a deeper understanding of how these elements influenced patient decision-making and clinical interactions.

Embodiment highlighted the importance of physically moving through the space to uncover lived experiences. By walking through the clinic, participants not only observed but also felt and experienced the environment, allowing us to understand how they perceived and responded to their surroundings.

Rhythm reflected the pace of activities within the clinical setting. The rhythm fluctuated depending on patient flow, the nature of procedures, and interactions happening in the clinical environment. This variability provided insights into how the pace of the clinic impacted participants' experiences and interactions.

Auto-photography further empowered participants by allowing them to document aspects of their environment that held personal meaning. Rooted in self-perception (19), this method enabled participants to represent their realities freely, without being constrained by researcher-led frameworks. Photo elicitation added another layer to this inquiry. Photographs taken by participants served as prompts for dialogue, enabling me to explore the meanings, emotions, and memories they associated with their images.

## Potential challenges of the walking method and mitigation strategies

Although some authors have noted that the walking method can be lengthy and pose practical and ethical concerns (4), we did not encounter these challenges in our study. We ensured that the method was structured efficiently, balancing engagement with time considerations while maintaining participant comfort. Additionally, some argue that sensitive discussions during the walking method could lead to misunderstandings among participants, potentially affecting data quality (20). However, we minimised power imbalances by employing facilitators from the same ethnic groups to conduct workshops in their mother tongues and by aligning our approach with Hofstede's Cultural Dimensions Theory (21). This helped ensure cultural sensitivity, foster inclusivity, and enhance participant engagement.

## Conclusion

While we demonstrated the tool's application for a specific case study, the UniCDent toolkit is versatile and can be customised for various research inquiries that have a spatial dimension to it. By incorporating these immersive techniques, researchers can develop patient-centred care strategies and explore diverse dimensions of clinical environments. This participatory method has allowed us to navigate the complexities of patient experiences, offering valuable insights that can enhance outcomes and advance dental research across various contexts. Indeed, this participatory method with legs has allowed us to walk into the complexities of patient experiences, paving the way for improved outcomes and greater understanding in any healthcare setting.

## Data Availability

The original contributions presented in the study are included in the article, further inquiries can be directed to the corresponding author.
